# *In vivo* Microbiome Profiling of the Luminal and Mucosal Surface of the Duodenum Using a Cannulated Yearling Bovine Model

**DOI:** 10.3389/fvets.2020.601874

**Published:** 2020-11-09

**Authors:** Ricardo M. Stockler, Keah V. Higgins, Haley Hallowell, Erin S. Groover, Elizabeth M. Hiltbold, Benjamin W. Newcomer, Paul H. Walz

**Affiliations:** ^1^Department of Clinical Sciences, College of Veterinary Medicine, Auburn University, Auburn, AL, United States; ^2^Department of Biological Sciences, College of Sciences and Mathematics, Auburn University, Auburn, AL, United States; ^3^Department of Pathobiology, College of Veterinary Medicine, Auburn University, Auburn, AL, United States

**Keywords:** duodenal cannulation, GIT microbiome, *in vivo* microbiome, bovine microbiome, metagenomic analyses

## Abstract

The gut microbiome provides important metabolic functions for the host animal. Bacterial dysbiosis as a result of bacterial, viral, and parasitic gastrointestinal infections can adversely affect the metabolism, productivity, and overall health. The objective of this study is to characterize the commensal microbiome present in the lumen and the mucosal surface of the duodenum of cattle, as we hypothesize that due to metabolic processes and or host proprieties, there are differences in the natural microbiota present in the mucosal surface and luminal contents of the bovine duodenum. Duodenal lumen contents and mucosal biopsies were collected from six dairy crossbred yearling steers. A flexible video-endoscope was used to harvest biopsy samples *via* a T shaped intestinal cannula. In order to assess as much environmental and individual steer microbiota variation as possible, each animal was sampled three times over a 6 week period. The DNA was extracted from the samples and submitted for16S rRNA gene Ion Torrent PGM bacterial sequencing. A detailed descriptive analysis from phylum to genus taxonomic level was reported. Differences in the microbiome population between two different sites within the duodenum were successfully characterized. A great and significant microbiota diversity was found between the luminal and mucosal biopsy At the phylum taxonomic level, Firmicutes, and Bacteroidetes composed over 80% of the microbiome. Further analysis at lower taxonomic levels, class, family, and genus, showed distinct diversity and distribution of the microbiome. Characterizing the gastrointestinal microbiome *in vivo* is imperative. The novelty of this study is the use of live cattle undergoing customary husbandry allowing real-time analysis of the duodenum microbiome contributing to the literature with respect to the bovine duodenum microbiome.

## Introduction

The microbiota refers to the entire population of microorganisms that colonizes a specific location of the body, and includes bacteria, fungi, archaea, viruses, and protozoans (1). The gastrointestinal (GIT) population of bacteria, in particular, plays an important role in the dietary metabolism of the host, including nutrient metabolism and utilization. Disruption of intestinal microbiota homeostasis, termed dysbiosis, can occur as a result of bacterial, viral, and parasitic gastrointestinal pathogenic infections adversely affecting host metabolism, productivity, and overall health (2).

Enteric diseases are known to be one of the major contributors, along with bovine respiratory disease, to decrease in feed consumption, weight gain, reduction in milk production, in dairy cattle, and deaths of youngstock, resulting in severe economic losses in the dairy and beef industries (3). The impact of such diseases extends to human health *via* the increased use of antimicrobial medications, risk of development of antimicrobial resistance, and the potential microbial contamination of human food products. Diarrhea accounted for 57% of deaths in unweaned dairy heifers according to the most recent National Animal Health Monitoring System USDA 2010 survey (4). Likewise, beef producers attributed 16, 18, and 2% of overall mortality to digestive disease in calves <3 weeks old, calves older than 3 weeks old, and breeding age cattle, respectively (4).

In ruminants, specifically cattle, the composition of the rumen microbiota and its impact on health, nutrition, and host physiological parameters has been studied (4–8). As mentioned above, metabolism of nutrients is key in the symbiotic relationship between the host and the microbiota. The intestinal microbiota is generally responsible for breaking down and metabolizing complex carbohydrates. Specifically, in ruminants, the breakdown of carbohydrates and complex plant materials starts in the rumen with nutrient absorption extending from the forestomaches throughout the intestinal tract. Bacteroidetes and Firmicutes are among the primary metabolically-active bacteria with a critical role in breaking down plant wall compounds and host-derived carbohydrates, including particles attached to the mucins or chondroitin sulfates of the protective mucosal layer of the intestine (2, 9). Changes in the Firmicutes: Bacteroidetes ratio (F:B) has been demonstrated to affect energy uptake from the diet and energy expenditure, contributing to the development of obesity in pigs, mice, and humans (2, 10).

Several peer-reviewed studies have been undertaken to analyze the function and/or describe the GIT bacterial communities in different production animals. The studies were typically conducted in animals shortly after euthanasia, from samples collected at slaughterhouses, from animals reared in sterile laboratory environments, or from animals that received a known transplanted microbiota (11, 12). However, these studies have multiple limitations, such as: cost (example: laboratory quality animals), sample collection method in the live animal, and complete loss of a production unit due to euthanasia and not harvesting for human consumption. Another factor known to influence the outcome of studies of the microbiota is the potential for disruption of the commensal microbiota through dietary changes, infection, and/or inflammatory processes. Additionally, it has been shown that tissue death alters bacterial populations (13, 14).

A full understanding of the GIT microbiota in cattle is still unrealized. While the characterization of the ruminal and fecal microbiota and its impact on bovine health and production has been previously investigated, the majority of studies examined only intraluminal samples harvested post-mortem (15, 16). To date, the bovine mucosal-associated microbiota has not been characterized, particularly in the live animal. This is relevant due to most metabolically-active processes occurring at the mucosal interface. The authors hypothesize that under normal husbandry, the luminal- and mucosal-associated microbiota of the bovine duodenum will differ significantly in their overall composition, as well as in their respective proportion. The purpose of this study was to provide a detailed analysis of the enteric mucosal microbiota *in vivo* through the use of serial mucosal biopsy and luminal samples collected endoscopically through a transabdominal-duodenal cannula surgically fitted in yearling cattle.

## Materials and Methods

### Animals

The study was conducted at the Auburn University College of Veterinary Medicine following approval of all procedures by the campus Institutional Animal Care and Use Committee (PRN 2015-2676). Six dairy, crossbred, healthy steers ~12 months of age and having an average body weight of 249 kilograms (range: 240–277 kilograms) were selected for inclusion in this study. All the study animals were housed in a one-acre pasture and followed a strictly controlled diet consisting of one flake of bermudagrass hay and five pounds of soy hull pellets per head twice daily, and fresh water ad libitum.

### Cannulation Model Technique

Three months prior to sample collection, the animals enrolled in the study had a T-shaped 1-inch intestinal cannula^1^ surgically fitted in the duodenum as previously outlined by Komarek (17). Briefly, with the animal standing and restrained in a livestock chute, analgesia of the right paralumbar fossa was achieved by regional infusion of 2% lidocaine hydrochloride. A standard laparotomy was performed followed by exposure of the pylorus to allow visualization and exteriorization of the duodenum. Approximately 6 cm aborad to the pylorus, a 5-cm anti-mesenteric incision was made in the duodenum. The duodenal cannula was inserted through the enterotomy site and the duodenal incision was closed over the cannula using an inverted closing pattern. A 15-cm incision in the body wall was then made caudoventral to the last rib in order to exteriorize and secure the duodenal cannula to its final location. The laparotomy incision was then closed using routine methods. A 7.5-cm rumen cannula^2^ was also surgically fitted in the rumen as previously described in the literature at the same time as duodenal cannulation for a concomitant rumen microbiota study (18). Post-operative treatment consisted of ceftiofur hydrochloride^3^, as an antibiotic, administered subcutaneously (2.2. mg/kg) once daily for 5 days and meloxicam^4^, as an antiflammatory, administered orally (1.0 mg/kg) once daily for 5 days. A 3-month recovery period was observed following surgery to allow complete healing of the surgical sites, ensure appropriate drug withdrawal periods were met, and provide research animals a consistent diet prior to study initiation and sample collection. Following the recovery period, all cattle were housed in the same pasture throughout the length of the study without fence-to-fence contact with other animals, and were fed a diet that remained consistent throughout the sample collection period.

### Sample Collection

In order to provide consistency and assess potential variation due to individual, environmental, and bacterial factors, each animal was sampled three times over a 6-week period. The order of sample collection was randomly assigned and is shown in Table 1.

**Table 1:** Timeline for sample collection.

**Calf ID**	**Week 1**	**Week 2**	**Week 3**	**Week 4**	**Week 5**	**Week 6**
69	X			X		X
70	X		X		X	
71	X		X		X	
7		X	X			X
10		X		X		X
50		X		X	X	

For sample collection, each individual calf was haltered and restrained in a livestock chute. The duodenal cannula was opened by manually removing the compression plug. A sterile 20-cm Foley urinary catheter was inserted completely through the cannula aborally to facilitate the collection of 0.5 to 1 mm of duodenal contents; these samples were designated as lumen contents samples. Next, a flexible video-endoscope^5^,^6^ was inserted through the cannula and advanced aborally 51.1 cm on average (range: 35–70 cm). Three mucosal biopsy samples, with a total average weight of 14.7 grams (range: 0.33–26.4 grams), of the mucosal surface were taken from each animal at each designated collection time point. All samples were placed in 750 μl of RNAlater immediately after collection, to preserve RNA integrity during storage at 4°C until processed.

### Sample Processing

#### DNA Isolation

A total of 18 luminal samples and 18 mucosal biopsy samples were collected for analysis and subsequent sequencing. Isolation of DNA from all samples was extracted using a commercial kit (E.Z.N.A® Stool DNA, Omega bio-tek®, Norcross, GA) according to the manufacturer's guidelines for DNA extraction in tissue, using glass beads, and for fluid samples. The pathogen detection protocol allows rapid and reliable isolation of purified DNA using a combination of reversible nucleic acid-binding properties of HiBind® matrix and spin column technology to allow the elimination of humic acid, polysaccharides, phenolic compounds, and enzyme inhibitors. The extracted DNA was eluted into 100 μl of sterile elution buffer and stored at −20°C until the time of DNA sequencing and bioinformatics analysis.

#### 16S rDNA Sequencing and Bioinformatic Analysis

The bacterial microbiome was analyzed using 16S rRNA gene V4 variable region PCR primers 515/806 in a single-step 30 cycle PCR using a commercially available kit^7^,^8^ following the protocol outlined by (33). Sequencing was performed on an Ion Torrent PGM (Personal Genome Machine) following the manufacturer's guidelines and processed using a proprietary analysis pipeline at MR DNA laboratory.

Sequences were de-multiplexed and sequence adaptors were removed prior to QIIME analysis (19). Bacterial composition was assessed using the Quantitative Insights into Microbial Ecology (QIIME) suite, QIIME2 version 2019.4. Reads were filtered for length and quality and chimeras were removed. Sequences were clustered into operational taxonomic units (OTUs) with a 97% identity threshold. Taxonomic assignment was performed using BLASTn classifier (trained by the SILVA database, release version 132) (20). OTUs with an abundance below 20 and present in less than five samples were not included in the downstream analysis. Remaining OTUs were consolidated into an OTU network for all individual samples using QIIME2 and this was imported into RStudio for downstream analysis.

#### Data and Statistical Analysis

Individual samples from each group were used to assess microbial abundance and variation for both sampling strategies. Alpha diversity was assessed through rarefaction graphs constructed with QIIME2. Relative abundance was used to calculate means and standard deviations of each group at each time point using the statistical program R (21). Using the RStudio statistical platform, *t*-tests were performed to identify significant difference in relative abundance of microbial taxa. Non-metric multidimensional scaling (nMDS) ordination was generated in RStudio using the *vegan* package (22). To generate the nMDS, raw bacterial hits were used to compute a sample dissimilarity matrix using the Bray-Curtis dissimilarity index. This matrix was then used to compute an ordination of the samples in two dimensions. The *vegan* package was also used to calculate Shannon's Diversity Index scores. Then, the Pielou's Evenness Index was calculated by dividing the Shannon's Diversity Index score by the log of unique species amount. Significance reported for any analysis is defined as *p* < 0.05.

## Results

After rigorous quality sequence curation, 1,444,966 sequences were parsed and then clustered. A total of 1,434.061 sequences identified within the Domain Bacteria were utilized for final microbiota analyses. The average reads per sample was 19,917.

**Figure 1:**
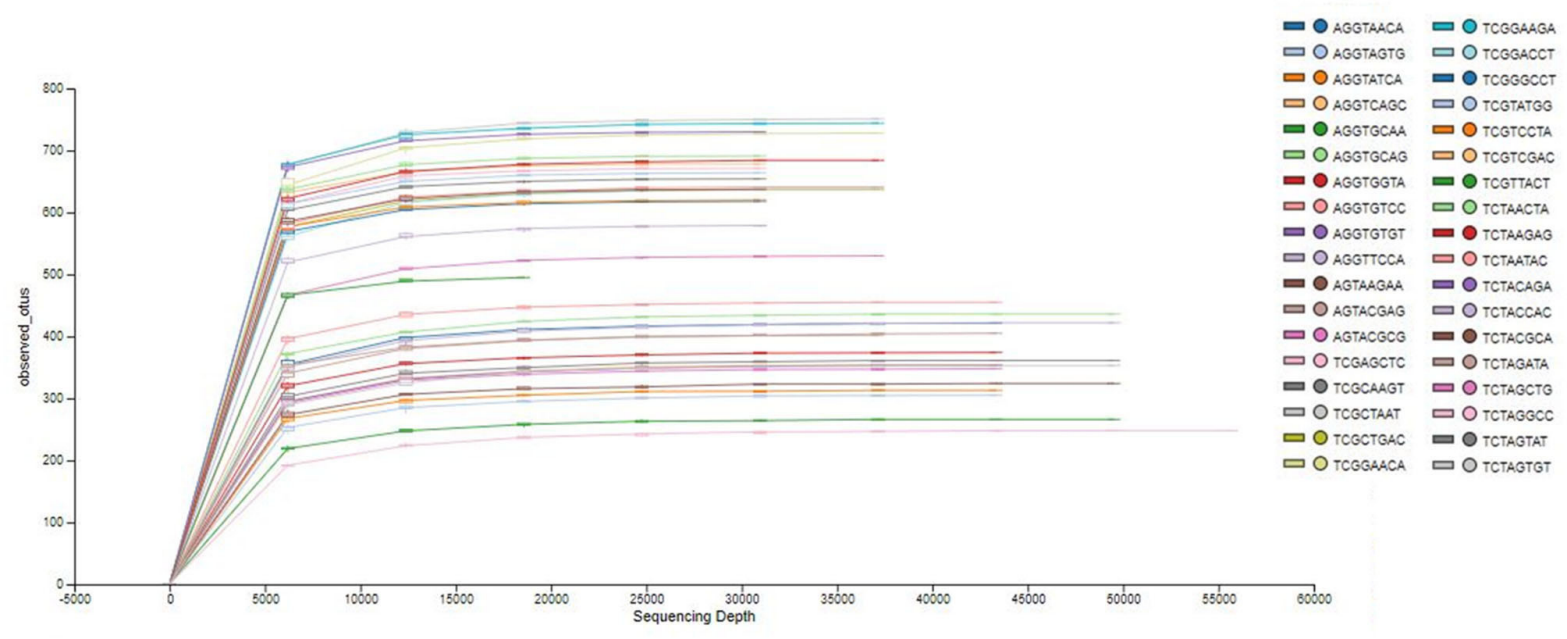
Phylogenetic rarefaction curves estimating species richness. The rarefaction curves produce smoother lines facilitating full dataset comparison by reaching a clear asymptote.

The analysis of the bacterial diversity is a function of sequencing effort and represented as individual samples by the color-coded lines. The positive assessment of richness for each sample collected is determined by the fact that each color-coded line achieved its maximum peak and plateau consistently with each other signifying adequate depth of sampling and alpha diversity (Figure 1).

**Figure 2:**
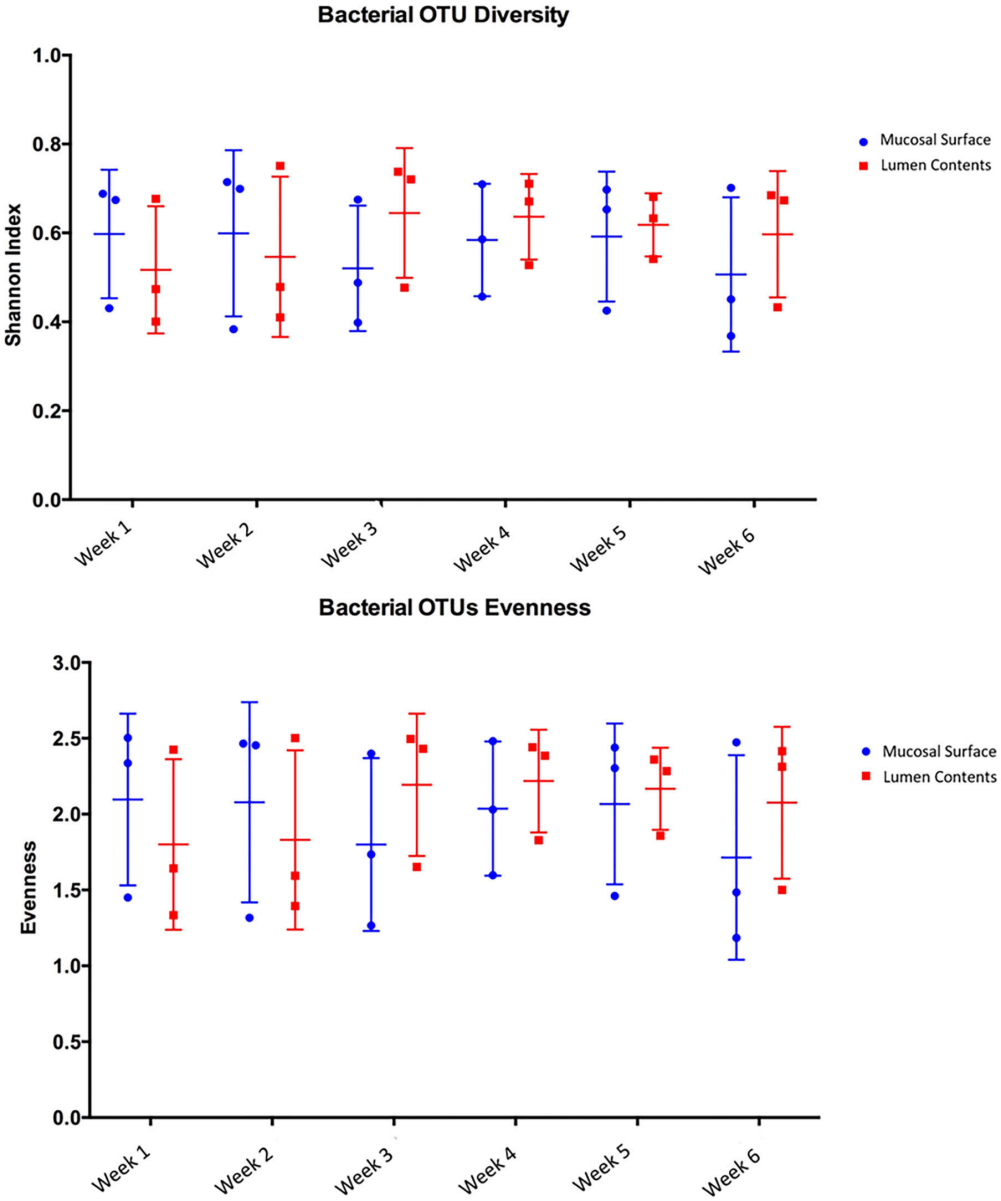
Comparison of bacterial OTU's Shannon index diversity and Pielou's evenness for the mucosal surface and lumen contents for each week sampled.

Species richness between the two locations, mucosal surface and lumen contents, were measured using the Shannon-Wiener Index, while evenness was measured utilizing Pielou's Evenness Index (Figure 2). Throughout the experiment, minimal change was observed in the diversity and evenness within the microbiota for both locations sampled. This is confirmed by the lack of statistical significance of the Shannon index reporting a *p*-value equal to 0.49 for the mucosal surface samples and 0.64 for the lumen contents, and for the evenness trend at 0.59 and 0.54 for the mucosal surface and the lumen contents, respectively.

**Figure 3:**
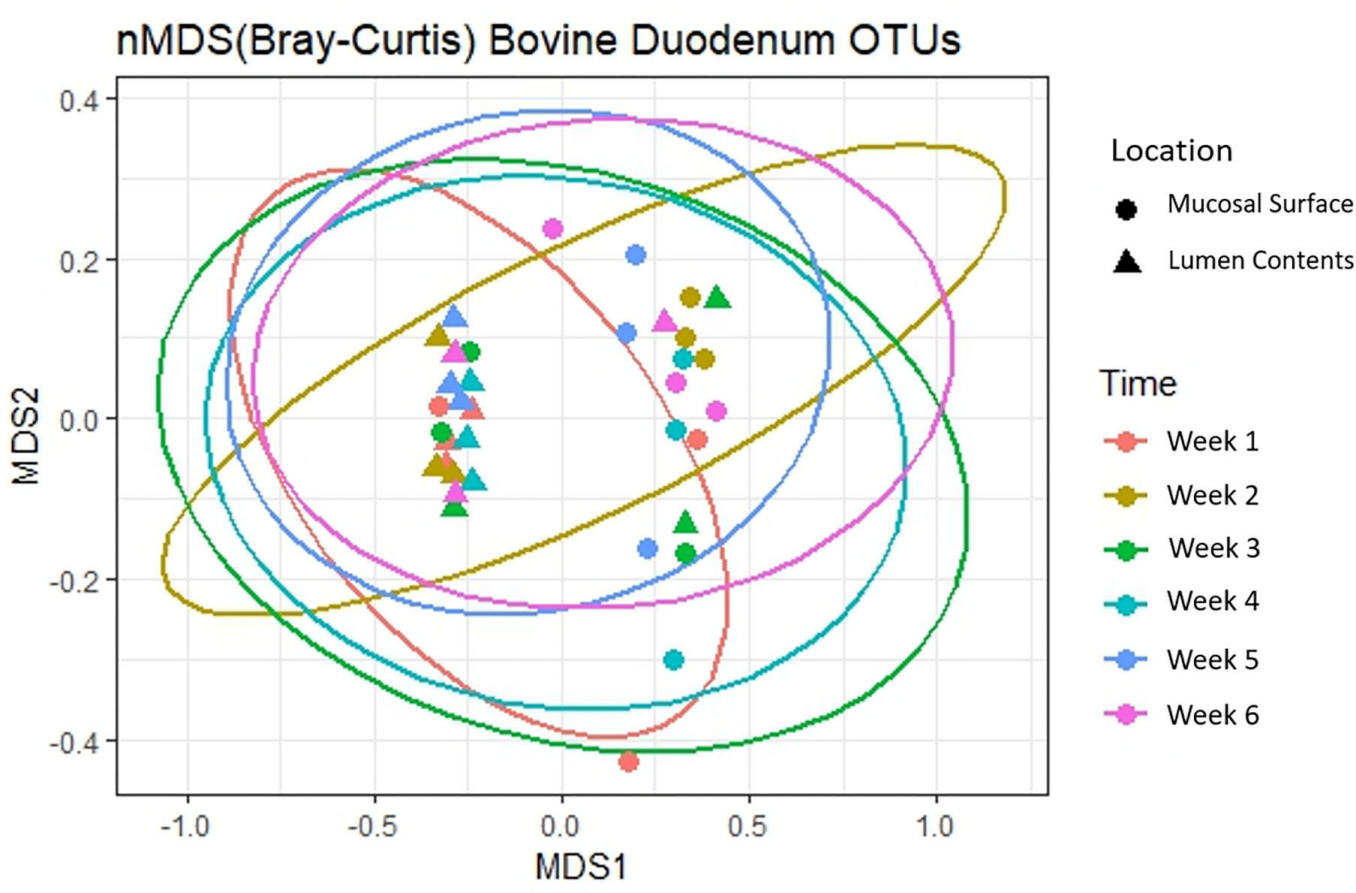
Multidimensional scaling plot (MDS) of bacterial lineages in the mucosal surface and lumen contents.

Next, to determine the amount of dissimilarity seen in the microbiota associated with the lumen and mucosal surface, an nMDS ordination plot utilizing a Bray-Curtis dissimilarity index was generated (Figure 3). Figure 3 demonstrates a clear separation of samples in the ordination plot, suggesting the microbiota between the two locations are dissimilar to each other as displayed by two distinct clusters of the same samples.

**Figure 4:**
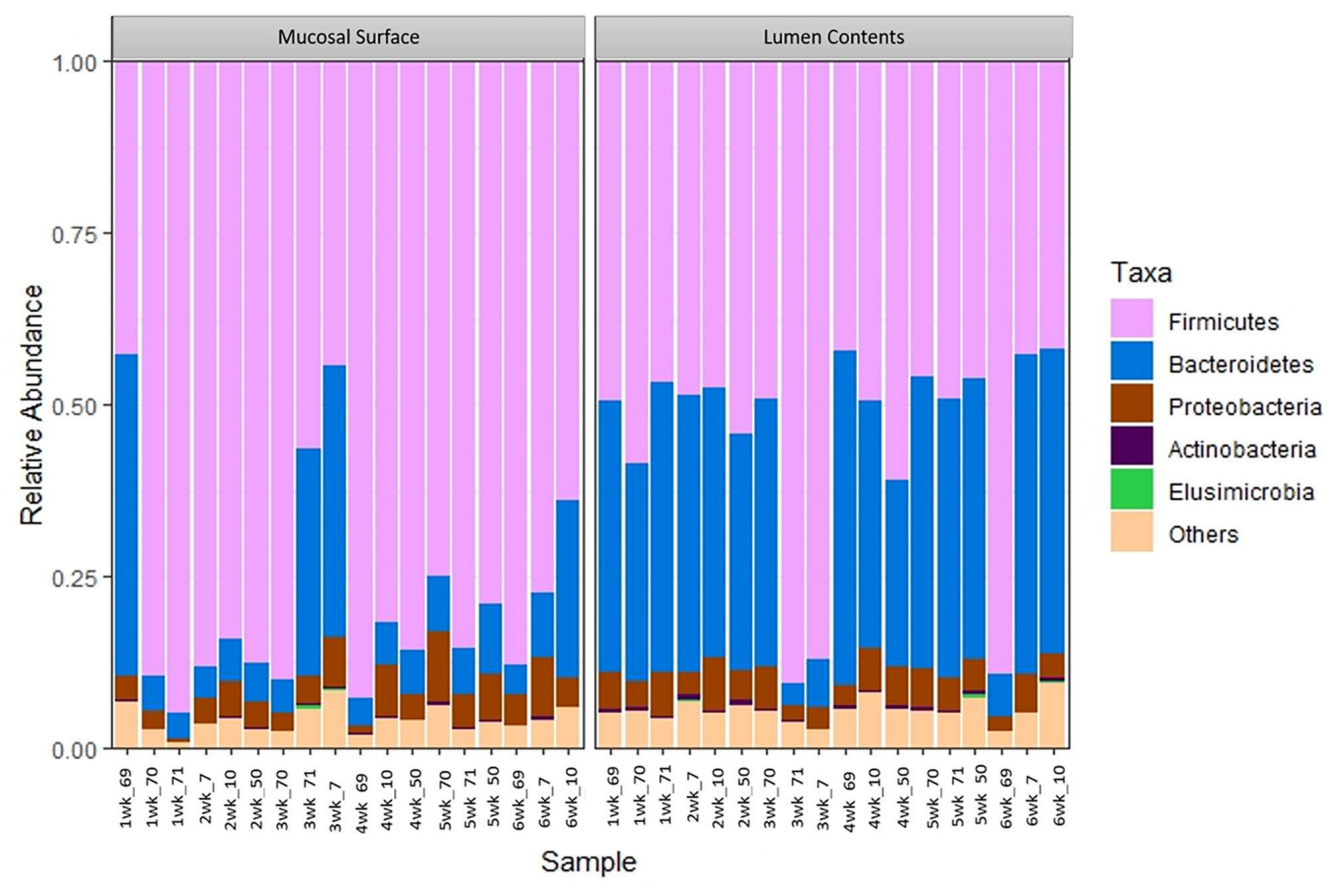
Bacterial phylum. Stacked bar chart representing the abundance of the top five phyla microbiota in the mucosal biopsy surface and lumen contents across the sampled weeks for each animal.

At the phylum level, Firmicutes (63%) and Bacteroidetes (21%) composed over 80% of the microbiome present in both sample locations. The relative abundance of Firmicutes was greater in the mucosal biopsy samples (75%) compared to the samples from the lumen contents (52%) for all cattle, whereas Bacteroidetes were mostly populated in the lumen contents (32 vs. 10%). The abundance of Proteobacteria and Actinobacteria were fairly similar, in total abundance, among the two locations (Figure 4). Overall, the F:B in the mucosal biopsy samples was significantly higher relative to the samples collected from the luminal contents especially on weeks 2, 4, and 5 (*P* = 0.005, *P* = 0.04, and *P* = 0.01 respectively), whereas, on weeks 1, 3, and 6 the statistical significance varied between *P* = 0.27 and 0.65.

**Figure 5:**
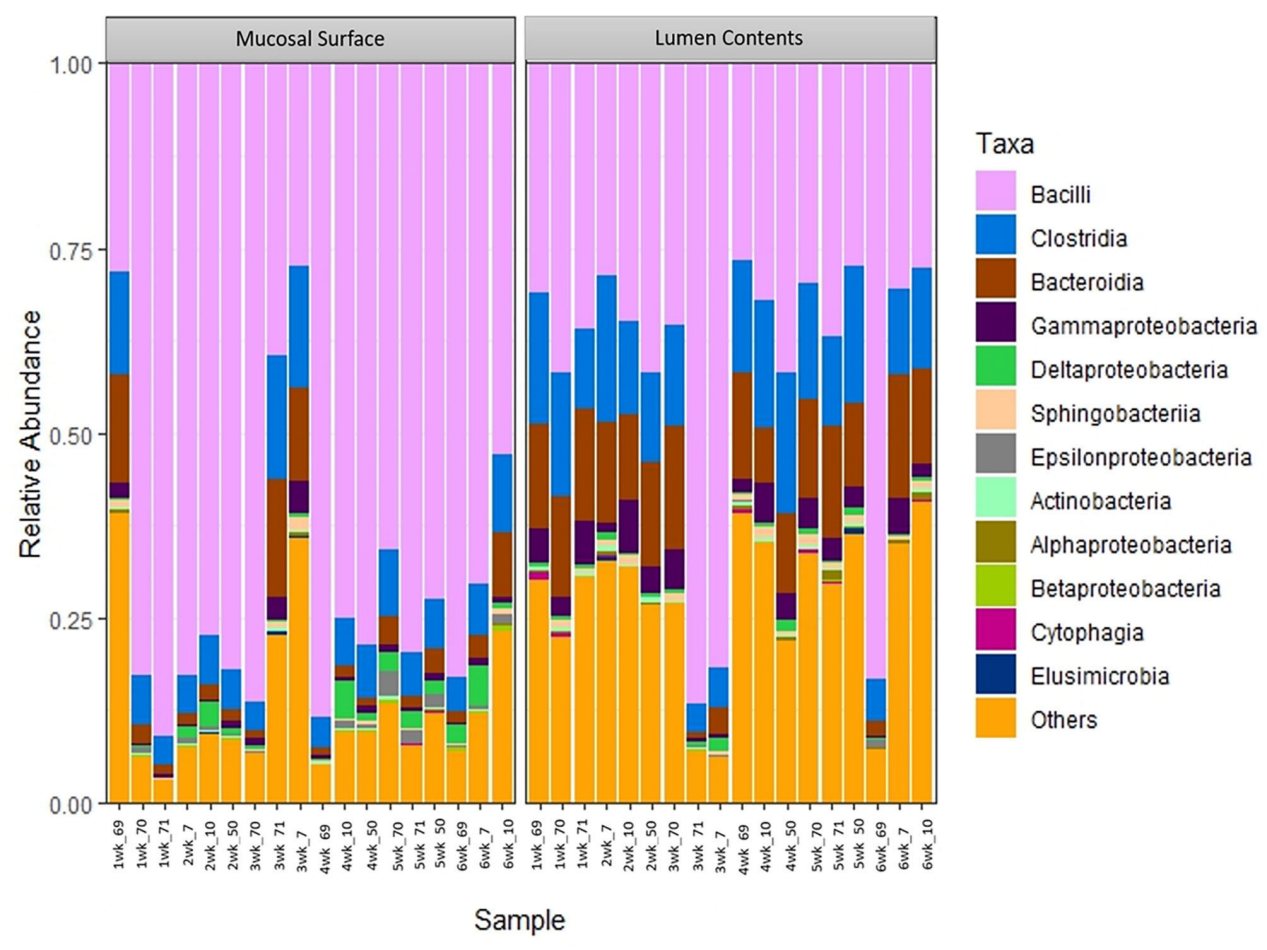
Bacterial class. Stacked bar chart representing the abundance of the microbiota at the class taxonomic level in the mucosal biopsy surface and lumen contents across the sampled weeks for each animal.

To further determine what populations are driving the dissimilarity between the two groups, the relative abundance at the taxonomic level of class was calculated (Figure 5). A significantly high abundance of Bacilli in the mucosal biopsy surface was observed (*P* = 0.02 – week 2, *P* = 0.001 – week 4 and *P* = 0.001 – week 5), whereas Clostridia and Bacteroidia were more abundant in the samples of luminal contents. Statistical significance was found during the same weeks as described above (Clostridia - *P* = 0.06, 0.001, and 0.02; Bacteroidia – *P* = 0.003, 0.03, and 0.002).

**Figure 6:**
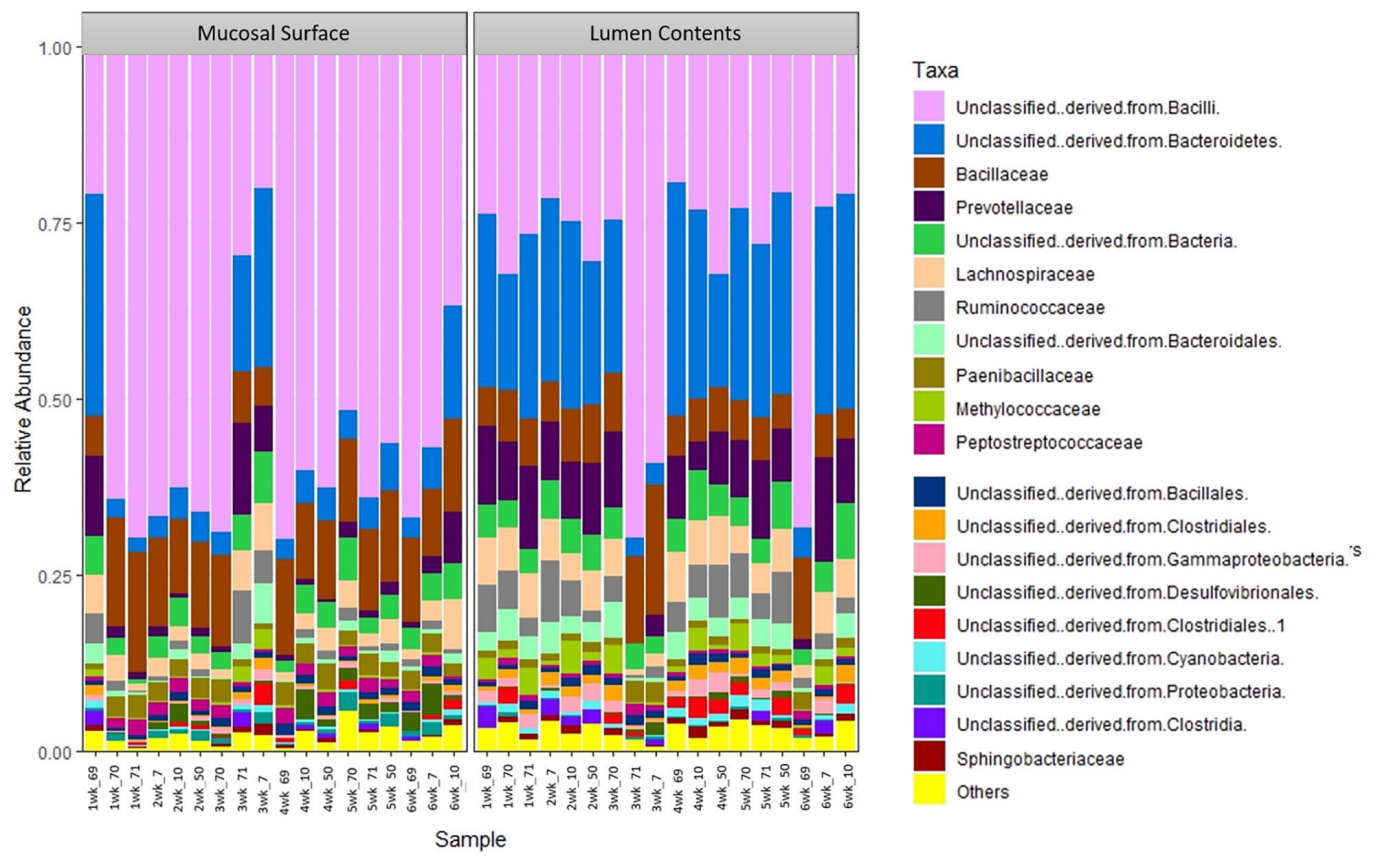
Bacterial family. Stacked bar chart representing the abundance of the microbiota at the family taxonomic level in the mucosal biopsy surface and lumen contents across the sampled weeks for each animal.

**Figure 7:**
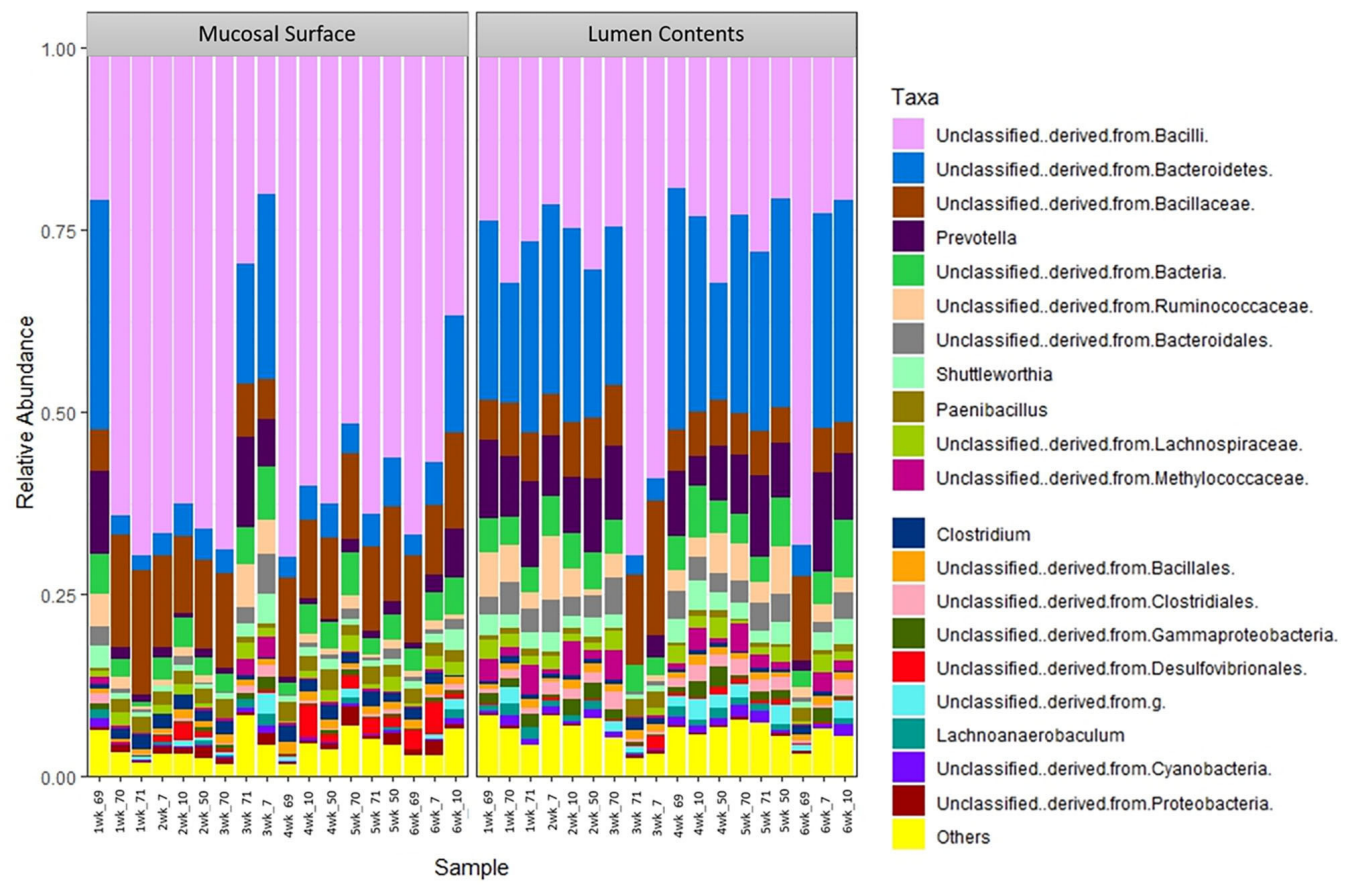
Bacterial genus. Stacked bar chart representing the abundance of the microbiota at the genus taxonomic level in the mucosal biopsy surface and lumen contents across the sampled weeks for each animal.

The same distribution between the two locations is seen at lower taxonomic level, at the family and genus, however it appears that the microbiota derived from the Bacteriodetes is predominant in the lumen contents, representing a shift from a mostly Clostridia abundance whereas no specific change or shifts were seen at the mucosal biopsy surface, as bacteria belonging to the class Bacilli predominates throughout (Figures 6, 7).

## Discussion

In this study, significant differences were observed between luminal and mucosal biopsy bacterial populations in the bovine duodenum. The method by which the duodenal mucosal biopsies were collected in this study is unique. A series of three endoscopic biopsy samples per animal per location were collected over a 6-week period *via* the surgically fitted duodenal cannula. This technique and approach allowed the collections to be executed in real time in the live animal undergoing normal husbandry.

Target gene sequencing using specifically Ion Torrent PGM 16S rRNA metagenomics method was used in this study. Genome sequencing using the 16S rRNA method is widely used among microbiome studies. This technique has a wide range of uses, including the characterization of a comprehensive variety of microbial diversity, taxonomical analysis, and species identification (23–25). Using a culture-based analysis, Creevey et al. (15), reported the existence of nine phyla in the rumen; in decreasing order of abundance the top four phyla reported were Firmicutes, Proteobacteria, Actinobacteria, and Bacteriodetes. In contrast, the main phyla found in duodenal samples in this study were Firmicutes, Bacteroidetes, Proteobacteria, and Actinobacteria in different abundance which varied by sample location. This indicates that although some phyla are conserved in different parts of the GIT, the exact abundance of the microbiome in different regions differs (5). Also, using 16 S rRNA pyrosequencing of the ruminal DNA, Jami, and colleague 2012, characterized and compared the rumen microbiota of cattle. This group suggested the existence of a core microbiome in the bovine rumen, and even though the variability was great, the authors demonstrated a high phylogenetic correlation among the described genera (7). In another study, the same researchers examined the rumen microbiome in lactating cows (6). The results were consistent with those of the first study, in which they demonstrated the presence of a core microbiome in the rumen. Specifically, they reported a bacterial population with 32% of the OTUs shared by at least 90% of the animals in the study and 19% of the OTUs common to 100% of the animals. Similarly, in the study reported here the commensal duodenal microbiota is also represented by a core microbiome with variability; with bacteria belonging to the phyla Firmicutes and Bacteroidetes representing 80% of the bacteria phylum present. However, the F:B in the mucosal biopsy samples was significantly higher relative to samples collected from the lumen. The same similar pattern was seen at the other taxonomic levels.

A study in swine used a similar method to successfully compare microbial populations in the mucosa and luminal microbiota in the colon of pigs, with and without dysentery, at necropsy (26). The authors demonstrated significant differences in the microbiome population of the gastrointestinal tissue and luminal ingesta between diseased and not diseased pigs. Furthermore, they also reported, at the genus level, the colonic bacterial population itself had changed in pigs with dysentery for both mucosal and luminal samples whereas a different population (Clostridiales, Erysipelotrichales, and Fusobacteriales) was seen in the luminal samples only. Those findings were comparable to the current study which demonstrated significant differences of the commensal population at all taxonomic levels between the mucosal biopsy and luminal sites in healthy animals. Thus, future studies of the microbiome must take into account population differences between sampling sites as, most likely, study results will vary as a direct effect of sampling location, technique, and potential disease processes.

In addition, De Rodas et al. (27), published the microbial profile from different anatomical sites of the GIT over time at different ages from farrow to finish using 16S rRNA V4 region sequencing with Illumina MiSeq. The group was able to observe shifts in the microbiome as the animals aged, as well as a positive correlation between several bacteria at the genus level and pig weight (27). In contrast, while the current study found a highly diverse population between the duodenal mucosa and lumen, a significant change in the microbiome profile over the 6-week sampling period was not observed, suggesting the duodenal microbiome is relatively stable over a short period of time. Microbiome studies of longer duration in cattle would be valuable to determine the impact that aging, diet, and other factors have on the microbiome profile. The commensal microbiome plays an important role in its interaction with the immune system, allowing the host to distinguish commensal and pathogenic bacteria. The higher species abundance observed for the mucosal communities suggests their core importance metabolically and immunologically to the host. Results of the current study are consistent with a previous study characterizing the GIT microbiome of pre-weaned calves, where significant differences were found in the bacterial populations of the mucosal surface and luminal contents (28). In that study, the authors proposed that the core metabolically active epimural bacterial population may survive mucosal immune defense mechanisms, and may be crucial for priming the host mucosal immune system. Therefore, the understanding of the commensal microbiota in different parts of the host, *in vivo*, is imperative (14).

The results of the current study showed the ratio of Firmicutes and Bacteroidetes in the duodenum mucosal biopsy samples were significantly higher relative to the samples collected from the lumen. This is consistent with previous reports that have analyzed F:B in mice and humans, where imbalances in the ratio in the GIT has been demonstrated to affect obesity and the capability of the host to harvest energy (2, 10). The microbiome present in obese hosts demonstrated greater capacity to harvest energy from the diet. Therefore, obesity in the host was supported and even exacerbated by the imbalanced bacterial populations (29, 30). Similarly, a correlation between pig weight and bacterial profiles has been demonstrated further supporting the idea that the microbiome is not an incidental finding, but an active player in the host's metabolism and health (27).

Using terminal restriction fragment length polymorphism (T-RFLP) analysis and quantitative PCR (qPCR) in conjunction with a clone library, Reti et al. analyzed and examined the bacterial communities associated with mucosa and within digesta throughout the intestinal tract of beef cattle (31). In their study, jejunal mucosal-associated bacterial communities consisted of mainly Proteobacteria, and differed conspicuously from those in the ileum and large intestine and mucosa-associated populations of the ileum, cecum, and descending colon where Firmicutes was the primary phylum identified. In contrast with the results presented in this manuscript, Proteobacteria were only the third most common phylum observed in both the mucosal biopsy and luminal samples representing ~6% of the population.

The authors speculate that the difference seen between the current study and the one published by Reti, is 3-fold. One, due to the sequencing method used, as Ion Torrent is more accurate the T-RFLP, two, due to dietary differences and lastly the methodology used to collect samples. Reti et al., collected the study samples at slaughter vs. *in vivo* and it is possible that Proteobacteria proliferates faster post-mortem and thus slaughter samples do not accurately reflect the *in vivo* populations.

The ruminant gastrointestinal microbiome grants many physiological and unique functions that are considered essential to maintain overall homeostasis. Significant differences in the bacterial populations of the lumen and mucosal surfaces of the bovine duodenum were identified in this study. This is consistent with other mammalian GIT microbiota studies by characterizing the presence of the three dominant phyla, Firmicutes, Bacteroidetes, and Proteobacteria. Results of this study indicate the duodenal microbiota of cattle is composed primarily of Firmicutes and Bacteroidetes. A much higher abundance of Firmicutes was observed in the mucosal surface than the luminal contents, and such pattern was also observed at lower taxonomic levels. This result is not unexpected as the active and controlled metabolism is believed to occur at the mucosal level. An important finding of this work was that all sampled animals shared the same primary group of bacterial classes, family, and genus; however, their respective abundance was significantly different between the sample locations. It has been suggested that the aerobic region within the intestines might be related to the outcome of interactions with the gut microbiota, acting as an innate immune barrier to protect the mucosal surface from anaerobic bacteria, while being recognized as a signal to promote invasion by pathogens (32). This concept may explain the standardized differences in bacterial abundance when mucosal biopsy and luminal contents are contrasted. Facultative aerobic Firmicutes, which have colonized the mucosal surface, may have readily available oxygen from the host essential for bacterial survival or as an advantage to growth, whereas the anaerobic environment of the lumen perhaps benefits the survival or enhanced growth of the Clostridia bacterial class. This principle is also observed with the Bacteroidetes in the results of this study; a larger and significant concentration of this phylum of bacteria is observed in the lumen vs. the mucosal surface.

Characterizing the gastrointestinal microbiome *in vivo* is imperative. This study documents the presence of significant different compositions of the bacterial populations in two distinct locations of the duodenum in live cattle undergoing normal and expected husbandry. This novel approach is crucial as many metabolically and biochemical changes in all body tissues are believed to be altered upon death (13). While this study demonstrates the differences in bacterial populations in different sites within the bovine duodenum and increases the understanding of the bovine duodenum microbiome, characterization of population differences between mucosal and luminal microbiota in different areas of the gastrointestinal tract remains to be described.

## Data Availability Statement

The datasets generated for this study can be found in online repositories. The names of the repository/repositories and accession number(s) can be found below: NCBI BioProject [accession number: PRJNA667247].

## Ethics Statement

The animal study was reviewed and approved by the IACUC at Auburn University PRN#2015-2676.

## Author Contributions

RS: designed, performed the experiment, and wrote the manuscript. EH, KH, and HH: bioinformatics and data analysis. EG: methodology development and sample collection. BN: major professor, directed the overall project and manuscript original drafting, review, and editing. PW: major professor, study's conceptualization, methodology, and surgical assistance. All authors contributed to the article and approved the submitted version.

## Conflict of Interest

The authors declare that the research was conducted in the absence of any commercial or financial relationships that could be construed as a potential conflict of interest.

## Footnotes

^1^Ankom® Open Intestinal Cannula, ANKOM Technology, Macedon, NY USA.

^2^Bar Diamond® INC. #8C, Parma, ID USA.

^3^Excenel® RTU EZ, Zoetis US Animal Health, Parsippany, NJ USA.

^4^Meloxicam 15 mg, Cipla USA, Inc., Miami, FL USA.

^5^Fujinon EV-450 LP5/23 with a Fujinon 4400 light source.

^6^E.Z.N.A® Stool DNA & HiBind® matrix, Omega bio-tek®, Norcross, GA USA.

^7^HotStarTaq Plus Master Mix Kita, Qiagen® Valencia, CA, USA.

^8^www.mrdnalab.com, MR DNA, Shallowater, TX.
